# A macrophage-like biomimetic nanoparticle with high-efficiency biofilm disruption and innate immunity activation for implant-related infection therapy

**DOI:** 10.1016/j.mtbio.2025.101575

**Published:** 2025-02-14

**Authors:** Guoqing Wei, Tiantian Xiao, Yufeng Xi, Rong Ju

**Affiliations:** Chengdu Women's and Children's Central Hospital, School of Medicine, University of Electronic Science and Technology of China, Chengdu, 611731, PR China

**Keywords:** Biomimetic, Biofilm microenvironment, Nitric oxide, Immunomodulation, Implant-associated infections

## Abstract

The innate immune system's inactivation and microbial biofilm-induced antibiotic resistance are the main causes of implant-associated infections (IAIs), which frequently result in implant surgical failure. Refractory recolonization is the consequence of standard therapies that are unable to consistently suppress escaping planktonic bacteria from biofilm, thereby enabling IAIs to thrive. Here, we specifically designed a macrophage-like biomimetic nanoparticle (F/R@PM) for a biofilm microenvironment (BME), which was fabricated by coating the cell membrane derived from macrophage onto poly (lactic-co-glycolic acid) (PLGA) namoparticles (NPs) loaded with FOT (NO donor) and R837 (TLR7 agonist). After injecting F/R@PM into mice with implant-associated infections, it was able to selectively target macrophages through macrophage membrane proteins on its surface and effectively release FOT and R837. Then, FOT that spreads outside the cell could react with glutathione (GSH) in the BEM to rapidly produce a large amount of NO inside biofilms to destroy the biofilm and kill bacteria. At the same time, R837 would encourage macrophages to scavenge planktonic bacteria that had escaped biofilm disintegration through improved phagocytosis. Overall, this work shows that NO treatment and immunotherapy together have promising potential for the long-term and efficient control and eradication of IAIs.

## Graphical abstract

**Figure:**
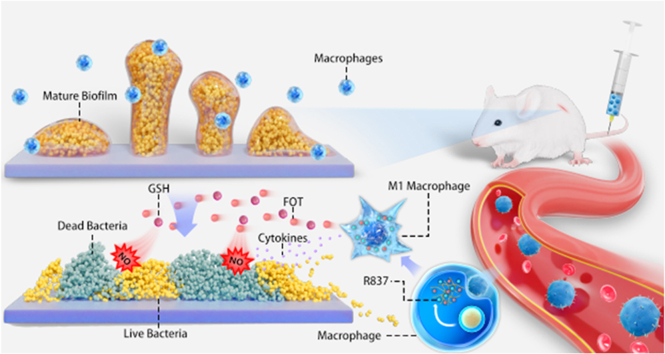

## 1 Introduction

Thousands of patients have benefited from implantable medical devices in their various forms, which have improved healthcare, therapy, and healing [1]. Nevertheless, the significant likelihood of implant-associated infections (IAIs) resulting from the development of microbial biofilms on the implant surface and adjacent tissues has emerged as the primary reason for the failure and recurrence of implant surgeries. This has become a major obstacle in the progress of implant surgery and a matter of concern for healthcare systems worldwide [[2], [3], [4]]. Due to their dense physical structure and extracellular polymeric substance (EPS), which is rich in polysaccharides and nucleic acids, bacterial biofilms are inherently resistant to antibiotics and impermeable to them [1,5,6]. As has been noted previously, bacterial cells included in biofilms can exhibit over 10^3^-fold resistance to antibiotics compared to their counterparts in planktonic form, which can lead to a chronic infection as well as higher rates of morbidity and mortality [[7], [8], [9]]. Additionally, by transitioning primary macrophages (M0) to an anti-inflammatory phase (M2) with reduced migration and suppressed bactericidal ability, the highly acidic and H_2_O_2_-rich biofilm microenvironment (BME) may inhibit the innate immune response, severely impeding biofilm clearance and even facilitating biofilm persistence at infection sites [[10], [11], [12], [13]]. The only available treatments for anti-IAIs presently include extensive debridement and implant replacement surgery, posing a significant burden on patients and society [7]. Even though previously developed anti-biofilm nanomaterials have been shown to inhibit bacterial adhesion or kill pathogens to prevent biofilm formation and avoid recalcitrant IAIs [[14], [15], [16]], it remains challenging to treat established IAIs due to the ineffectiveness of current nano-antibacterial strategies in destroying the complex extracellular mixture found in biofilms. In addition, the ability of planktonic bacteria to escape and survive in an immunologically suppressed milieu allows for the possibility of recolonization after the disintegration of bacterial biofilms [17]. Furthermore, latent bacteria can re-adhere and construct new biofilm structures when the external therapy is discontinued [18,19]. Since few of these currently available nanomaterials are involved in enhancing the local immune microenvironment of IAIs, it would be extremely important to develop a novel nanoplatform that can completely eradicate IAIs by stimulating proinflammatory immunity and disrupting mature biofilms.

Nitric oxide (NO), a well-known gas messenger molecule, plays a critical role in numerous physiological and pathological processes, including neurotransmission, immunological responses, and cardiovascular homeostasis [20,21]. Growing evidence in the field of microbiology indicates that relatively high NO levels function as cytotoxic and apoptosis-inducing agents [22]. Microbial proteins, DNA, metabolic enzymes, and outer membrane structures can sustain oxidative and nitrosative damage when NO is present alone or in conjunction with reactive nitrogen species (RNS), which are generated when NO combines with reactive oxygen species (ROS) [22,23]. Furthermore, by decreasing cyclic diguanylate (c-di-GMP), which enhances bacteria-surface attachment and subsequently biofilm formation, it has been reported that NO can penetrate deeply into biofilms to damage bacteria and effectively induce biofilm dispersal in *Pseudomonas aeruginosa, Staphylococcus aureus*, and other bacterial species [[24], [25], [26]]. Because of this, NO-based nanoparticles (NPs) have garnered a lot of attention because they may serve as a good substitute for treating persistent biofilm infections.

Another unresolved challenge lies in the elimination of the pulverized biofilm and planktonic bacteria that emerge from the fragmentized biofilm [27]. These bacteria may result in the recurrence of IAIs or the occurrence of acute infection due to the defective innate immunity surrounding implants [27]. As a result, activating local macrophage-associated immunity is a viable strategy for the complete eradication of IAIs by entrapping the pulverized biofilm and releasing planktonic bacteria, along with NO's anti-biofilm and bactericidal properties. Because immunoagonists (Fe_3_O_4_ nanoparticles [28], toll-like receptor [TLR]3 agonists [29], TLR7 agonist [30]) can activate proinflammatory immunity, which has been widely used in the field of tumor immunotherapy, a nanocarrier is thus required to simultaneous delivery NO donors and immune activators to obtain a better conquer IAIs effect. Furthermore, the membrane of macrophages that specifically target BME was coated on the NPs to increase the cumulative amount of drug-carrying NPs at BME in vivo.

**Fig. 1:**
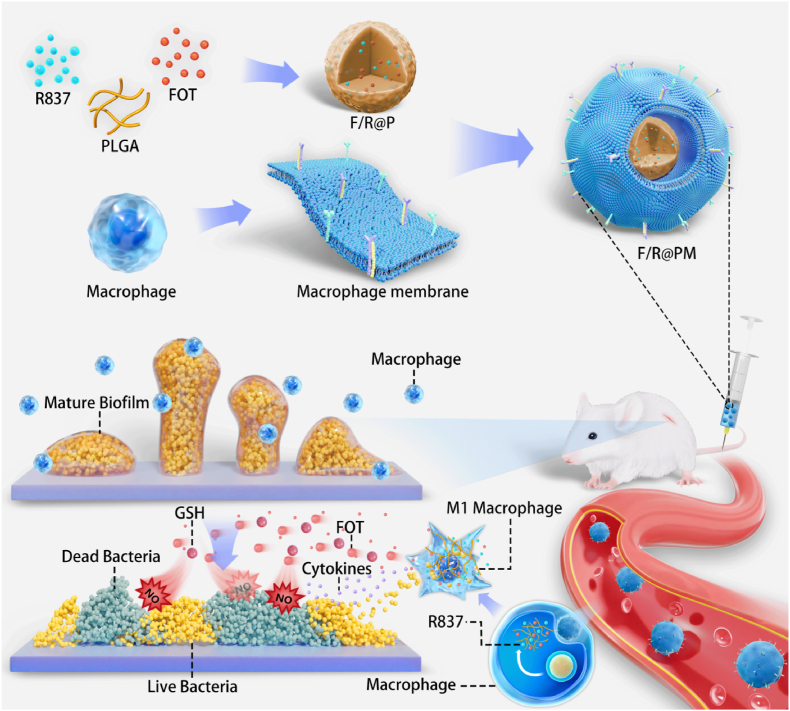
Schematic of F/R@PM fabrication and its in vivo treatment for implant-related infections.

Here, we designed a biomimetic nanoparticle (termed as F/R@PM), aiming to precisely target therapy IAIs. FOT (NO donor) and R837 (TLR7 agonist)-loaded poly (lactic-co-glycolic) acid (PLGA) NPs were coated with the macrophage cell membrane (MM) to create F/R@PM, which was inspired by the unique physiological shifts that occur after infection (Fig. 1). This enabled inflammatory homing and multi-mechanism combination therapy of the infection site. On the one hand, with the specific uptake by macrophages for the membrane components, FOT and R837 could be released from NPs due to the low-acid environment of lysosomes (pH 5.0). The released FOT would react with glutathione (GSH) in the milieu to continuously generate NO in the biofilm matrix after it spreads outside the cell. This process has the potential to efficiently eliminate bacteria and destabilize the rigid biofilm. However, the nuclear factor kappa B signaling pathway would be primarily activated by the released R837. This effectively achieved proinflammatory polarization of macrophages, which in turn reversed the immunosuppressive milieu surrounding the biofilm, resulting in the elimination of the pulverized biofilms and the dispersal of floating bacteria. In this investigation, we validated the potential of F/R@PM in combating implant-related biofilm infections by effectively integrating planktonic immune suppression and biofilm scavenging to eradicate recalcitrant effects. This treatment strategy for all-stage IAIs offers a novel paradigm for current treatment modalities and has the potential to have significant clinical applications.

## 2 Results and discussions

### 2.1 The characterization and production of biomimetic NPs

**Fig. 2:**
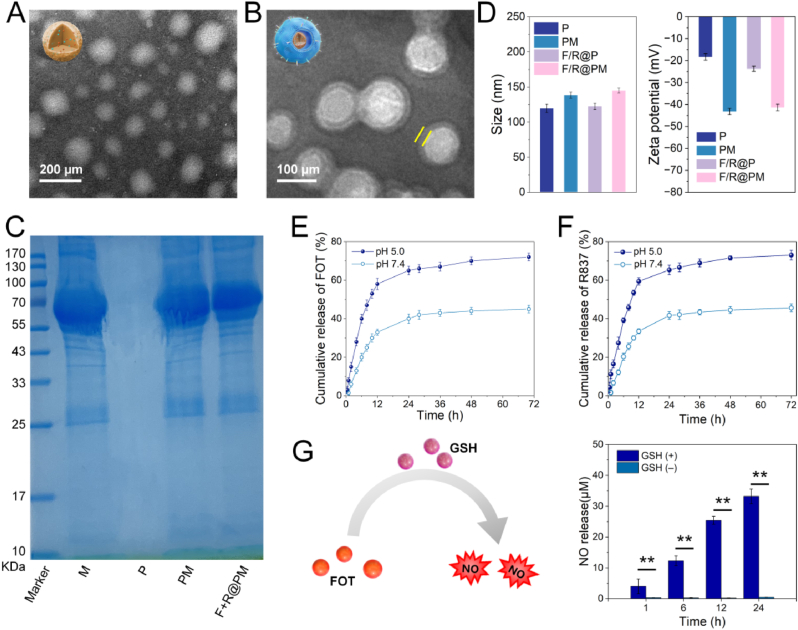
Characterization of the macrophage-like biomimetic nanoparticle F/R@PM. TEM images of (**A**) F/R@Pand (**B**) F/R@PM. (**C**) Protein expression profile of fresh macrophage cell membrane (M), P, PM, and F/R@PM were separated by SDS-PAGE followed by Coomassie brilliant blue staining. Marker, molecular weight in kilodaltons. (**D**) The average hydrodynamic diameter and surface zeta potential of P, PM, F/R@P, and F/R@PM as measured by DLS (n = 3 per group). (**E** and **F**) In vitro drug release profiles of F/R@PM under varied pH values (n = 3 per group). (**G**) Schematic diagram of GSH-mediated NO release from FOT (left) and NO gas release profiles of F/R@PM with or without GSH under pH 5.0 (n = 3, ∗∗*p* < 0.01) (right). (For interpretation of the references to color in this figure legend, the reader is referred to the Web version of this article.)

Using a nanoprecipitation method, immunoregulator R837, GSH-responsive NO donor FOT, and clinically utilized PLGA polymer were combined to create F/R@P NPs. F/R@P NPs showed a monodispersed spherical structure, as observed using TEM scans (Fig. 2A). The result of the UV–visible absorption spectrum provided additional evidence that the F/R@P NPs were successfully synthesized (Fig. S1). FOT and R837 were fully contained in the PLGA core, as evidenced by the moderate absorption peaks at 254 and 318 nm that the F/R@P NPs dimethyl sulfoxide solution displayed in comparison to PLGA. HPLC was used to assess the loading content (LC) and EE of FOT and R837. The results showed that the LC was approximately 10.2 % and 4.1 %, the EE was approximately 59.8 % and 41.0 %, respectively (Fig. S2). Additionally, using a previously described procedure [31], MM vesicles were extracted from mouse mononuclear macrophage leukemia cells (RAW264.7) and coextruded with F/R@P NPs to create MM-coated F/R@P NPs (F/R@PM NPs), which enhanced the stability and targeting capacity of NPs. The TEM image's distinctive shell structure of the F/R@P NPs core surface indicated the effectiveness of the MM coating (Fig. 2B). Additionally, the SDS-PAGE experiment was used to investigate the membrane proteins. Both PM and F/R@PM contained more RAW264.7 proteins than the RAW264.7 membrane, indicating that membrane proteins persisted after physical extrusion (Fig. 2C). The presence of integrins α4 and β1, which are essential in targeting, as well as CD47, a critical cell surface marker that regulates macrophage phagocytosis, were confirmed by Western blot analysis. As seen in Fig. S3 The presence of CD47, integrin α4, and integrin β1 protein signals was confirmed in MMs, PM, and F/R@PM NPs, but not in P NPs. Compared to uncoated F/R@P NPs, the hydrodynamic diameter of F/R@PM NPs increased by approximately 22.2 nm. Successful encapsulation of F/R@P NPs was indicated by the zeta potential of F/R@PM NPs (−41.5 mV), which was significantly higher than that of uncoated F/R@P NPs (−18.4 mV) but similar to that of PM NPs (−43.3 mV) (Fig. 2D).

To study release behavior, we first investigated the stability of F/R@PM NPs dispersed in different media. As illustrated in Fig. S4, the F/R@PM NPs maintained a relatively consistent size in water and a medium containing 10 % FBS for 7 days after being stored at ambient temperature for an extended period. This suggests the stability of the NPs. Subsequently, the release kinetics of medications from F/R@PM NPs were examined in buffer solutions that stimulated the extracellular environment (PBS, pH 7.4) and intracellular lysosomal environment (ABS, pH 5.0) at 37 °C using HPLC. Following a 72-h incubation period, F/R@PM NPs released 45.0 % of FOT and 45.6 % of R837 at pH 7.4, but at pH 5.0, a greater release amount was observed, measuring 72.0 % of FOT and 73.0 % of R837 (Fig. 2E and F). These findings suggest that F/R@PM NPs can release drugs quickly after internalizing into the acidic endosomes of macrophage cells.

Using the Griess test, the concentrations of nitrite, an oxidative metabolite of NO, in PBS (with or without GSH) following co-incubation for 1, 6, 12, and 24 h were measured to study GSH-induced NO release from F/R@PM NPs (pretreatment in ABS for 12 h). As shown in Fig. 2G, F/R@PM released a significantly larger amount of NO when F/R@PM co-incubation with GSH (*P* < 0.01), while almost no NO is produced in the absence of GSH, suggesting that F/R@PM could effectively release NO when attacked by thiol-containing molecules.

### 2.2 Homologous targeting in vitro and in vivo

**Fig. 3:**
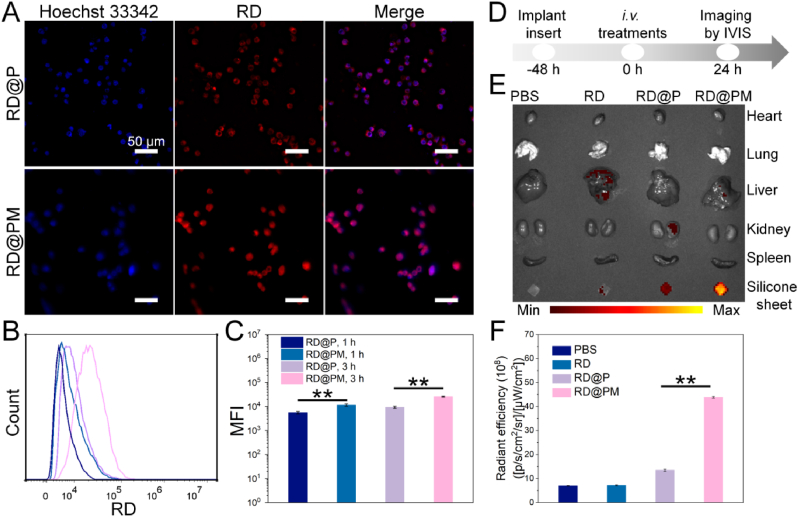
Cell uptake and targeting capacity of MP NPs. (**A**) Representative fluorescence images of RD@P and RD@PM internalized by RAW264.7 cells. (**B**) FACS results of cellular uptake of RD@P and RD @PM in RAW264.7 cells. (**C**) Quantification of cellular uptake of RD@P and RD@PM in RAW264.7 cells (n = 3, ∗∗*p* < 0.01). (**D**) The experimental scheme of drug targeting in vivo in the implant infection model. Representative ex vivo fluorescence images (**E**) and quantification analysis (**F**) for PBS, free RD, RD@P, and RD@PM accumulation in the silicone sheets and major organs of IAI mice at 24 h after intravenous injection (n = 3, ∗∗*p* < 0.01).

It is important to note that homologous targeting via macrophage membranes has a solid body of research behind it [32]. Consequently, we examine how homologous targeting by macrophage membranes facilitates endocytosis in this section. To investigate the time-dependent dynamics of cellular uptake after FM injection, we utilized RB-labeled PM NPs (RB@PM) and P NPs (RB@P). As shown in Fig. 3A, RB@PM NPs significantly outperformed RB@P NPs in RAW264.7 cells in terms of mean fluorescence intensity (MFI), suggesting that macrophage cell membrane components play a major role in NP endocytosis and cell-membrane modified NPs can deliver more therapeutic drugs into the target cell, resulting in better therapeutic effects. To assess MM-coated NPs' ability to target macrophages selectively, a novel experimental configuration was created in which RB@PM NPs were cultured with HUVECs. After 3 h of incubation, FM analysis showed that the red fluorescence intensity surrounding the HUVEC nucleus underwent a minimal alteration (Fig. S5A). The intensity of RB fluorescence in RAW264.7 macrophages or HUVECs was then measured using flow cytometry after the cells had been incubated with RB@P or RB@PM NPs. The results suggested a considerable increase in PM NP cellular uptake, primarily in the RAW264.7 cell line (Fig. 3B and C). In contrast, HUVECs showed a marked decrease in their ability to internalize nanoparticles (Fig. S5B). These findings thus offer substantial proof of the recommended MM-coated PLGA NPs’ potent macrophage-targeting efficacy.

Furthermore, we studied whether PM NPs could specifically target the implant site of mice infected with *S. aureus* in vivo. The experimental scheme is shown in Fig. 3D. First, the silicone sheet with *S. aureus* bacterial biofilm was implanted into the back of the mice, which induced implant-associated infection in mice and stimulated the aggregation of macrophages. After 1 day, PBS, free RD, RD-loaded P NPs, and PM NPs were injected intravenously. At 24 h after intravenous injection, the biodistribution of free RD and RD-loaded NPs in the silicone sheets and major organs of mice were investigated with IVIS (Fig. 3E and F). Ex vivo fluorescence imaging revealed a notable fluorescent accumulation in silicone sheets of the RB@PM group, with a 6.4-fold higher MFI compared to the free RD group and with a 3.4-fold higher MFI compared to the RB@P group (*P* < 0.01). Notably, strong signals were exclusively detected in the livers of free RB-treated mice, indicating rapid clearance from systemic circulation. However, a preferential accumulation in the silicone sheets of RB@PM-treated implant-associated infection mice was observed. This result further demonstrated that PM NPs can effectively prevent drug clearance and increase the targeted enrichment of drugs in infected lesions.

### 2.3 In vitro biofilm eradication assay of F/R@PM NPs

**Fig. 4:**
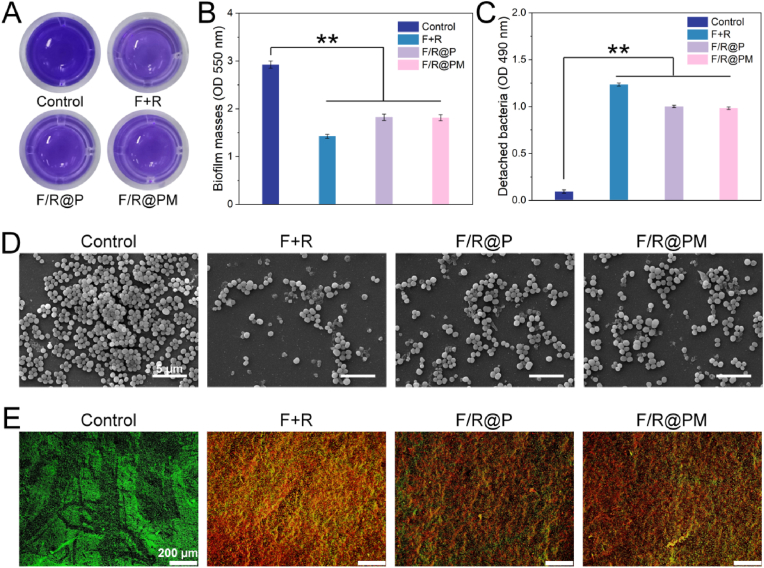
Antibiofilm properties of F/R@PM. (**A**) The photographs of *S. aureus* biofilm processed with different treatments and subjected to crystal violet staining. (**B**) Biofilm biomass of *S. aureus* biofilm after various treatments. (n = 3, ∗∗*p* < 0.01). (**C**) Detached bacteria biomass from *S. aureus* biofilm after various treatments. (n = 3, ∗∗*p* < 0.01). (**D**) Representative SEM images of *S. aureus* biofilm. (**E**) Representative fluorescence images of *S. aureus* biofilm stained with SYTO 9/PI. (For interpretation of the references to color in this figure legend, the reader is referred to the Web version of this article.)

Biofilms grown on 48-well plates were divided into four groups to verify F/R@PM disruption of the 3-dimensional biofilm barrier: control (PBS), F + R (FOT and R837), F/R@P NPs, and F/R@PM NPs (all nanoparticles were pre-incubated in ABS for 12 h). In a culture medium containing 10 mM GSH, the infection microenvironment was simulated. Biofilms were stained with crystal violet after treatment [18]. The result shows that the F + R, F/R@P, and F/R@PM groups effectively cause biofilm dissociation and integrity degradation in comparison to the control group (Fig. 4A). The F/R@P and F/R@PM groups do not significantly differ from one another, suggesting that their ability to destroy biofilms in vitro is equivalent. The quantitative results show that compared with the control group, F/R@P and F/R@PM groups evoke efficient biofilm dissociation and integrity destruction (*P* < 0.01, Fig. 4B). Additionally, bacteria that had separated from the biofilm were gathered and examined at 490 nm absorbance [33,34]. The findings demonstrate that FOT alone is sufficient to separate mature biofilms and release the bacteria that are contained inside them (*P* < 0.01, Fig. 4C). In addition, biofilms cultured on shims were dried and preserved for scanning electron microscopy (SEM) examination. The results showed that in comparison to the control group, the biofilms in the F + R, F/R@P, and F/R@PM groups had dissolved (Fig. 4D). Moreover, confocal laser scanning microscopy (CLSM) images (Fig. 4E) demonstrate this observation.

### 2.4 In vitro immunomodulation assay of F/R@PM NPs

**Fig. 5:**
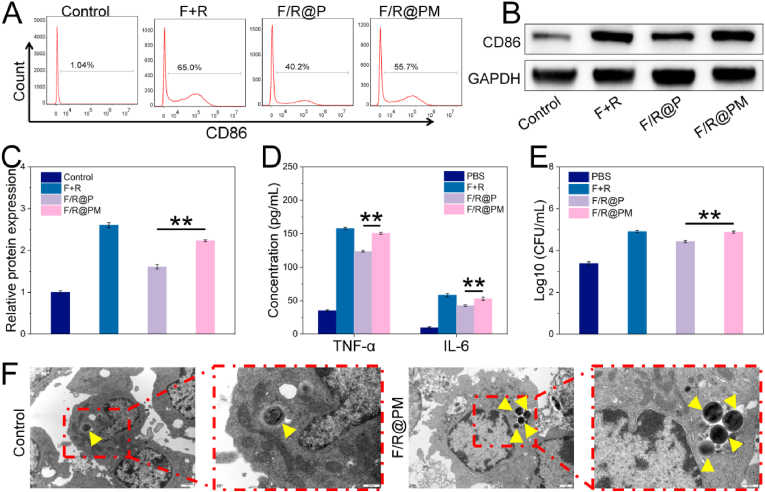
Immunomodulatory effects of F/R@PM on macrophages in vitro. (**A**) Typical scatter plots of macrophage surface markers CD86 (M1 macrophage marker) as detected using a flow cytometer. (**B**) Representative western blot results of the expression of CD86 protein after various treatments. (**C**) Corresponding quantitative analyses of western blot results (n = 3, ∗∗*P* < 0.01). (**D**) ELISA results of cytokines (TNF-*α* and IL-6) secreted by RAW264.7 in different groups (n = 3, ∗∗*P* < 0.01). (**E**) Counted results of phagocytized *S. aureus* by RAW264.7 treated in different conditions (n = 3, ∗∗*P* < 0.01). (**F**) Swallowed *S. aureus* was observed by using TEM. Yellow arrows indicate *S. aureus*. (For interpretation of the references to color in this figure legend, the reader is referred to the Web version of this article.)

Although FOT can effectively damage biofilms and eliminate most bacteria, IAIs could still recur. To further suppress IAIs, F/R@PM NPs were designed to target macrophages and induce their conversion to the M1 phase. The expression of TLR-7 in RAW264.7 was first verified (Fig. S6). And then following treatment with PBS, F + R, F/R@P, or F/R@PM, RAW264.7 macrophages were collected for flow cytometry analysis (Fig. 5A). The findings show that compared to the PBS group, the CD86 fluorescence intensity was considerably higher in the F + R, F/R@P, and F/R@PM groups. Moreover, the F/R@PM group exhibited higher CD86 fluorescence intensity than the F/R@P group, suggesting that F/R@PM markedly enhanced RAW264.7 cells’ M1 polarization. Using a western blot analysis, we further showed that F/R@PM significantly increased the expression of M1 macrophage molecular markers (CD86) (*P* < 0.01, Fig. 5B and C). Previous literature [35] states that phagocytosis and the release of inflammatory cytokines are the two primary mechanisms of macrophage innate defense against bacteria. Therefore, to detect macrophage polarization in upcoming immunoregulation investigations, these indicators were selected. An ELISA kit was used to measure TNF-α and IL-6 levels (Fig. 5D). F + R and drug-loaded NPs (F/R@P or F/R@PM) stimulation were found to upregulate TNF-α levels in comparison to the control group. Furthermore, compared with F/R@P, F/R@PM had a better upregulation effect. These results indicated that drug-loaded NPs could effectively activate macrophages to pro-inflammatory type and up-regulate the release of inflammatory factors (*P* < 0.01), and NPs coated with cell membranes have better effects than those uncoated due to the targeting action of cell membranes, showing better immunomodulatory ability.

Additionally, RAW264.7 was co-cultured with *S. aureus* in 3 pretreatment groups to validate the phagocytic activity of activated M1 macrophages. Following their isolation from lysed macrophages, phagocytized bacteria were counted by TEM examination and the spreading plate method, respectively. The phagocytic effects of the F + R, F/R@P, and F/R@PM groups were all improved; however, the amount of phagocytized *S. aureus* was lower in the F/R@PM group than in the F/R@P group (*P* < 0.01). There is a noticeable decrease in *S. aureus* in the PBS group (Fig. 5E and F). These results demonstrate that F/R@PM is a potent immunomodulatory drug that enhances the shift of M0 macrophages to the proinflammatory M1 phase. According to earlier studies, bacterial biofilms may cause macrophages to become polarized against inflammation, which would lower innate immunity locally [36]. F/R@PM can activate the antibacterial functions of macrophages, thus reversing biofilm-induced immune evasion.

### 2.5 F/R@PM eliminate IAIs in vivo

**Fig. 6:**
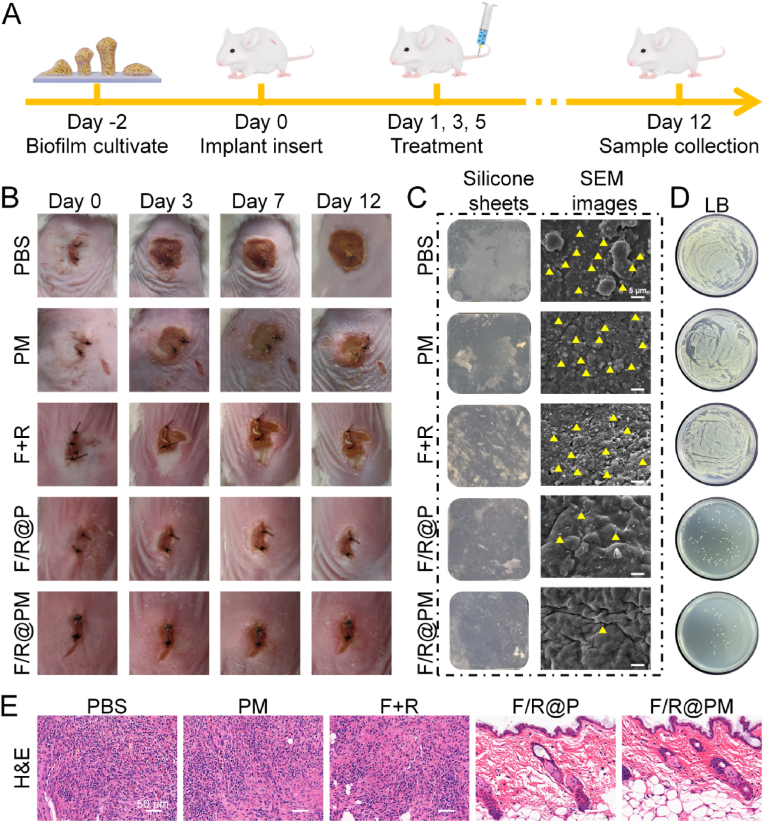
Effects of F/R@PM on IAIs in vivo. (**A**) Schematic diagram of the in vivo treatment procedure of the IAIs assay. (**B**) Photographs of mice after various treatments and wound healing condition. (**C**) Macroscopic photos and SEM images of Silicone sheets collected from each group. (**D**) Macroscopic photos of LB results of the ultrasonic oscillation solution. (**E**) H&E staining of the peripheral tissue after various treatments.

We investigated the antibiofilm potential of F/R@PM in vivo using a mouse IAI model because of its remarkable biofilm elimination ability in vitro. The evolution of the IAIs model and the related treatment and observation protocols are shown in the schematic design (Fig. 6A). Day zero was the day that the infection model was developed. To create the biofilm, the sterile silicone sheets were submerged in a suspension of *S. aureus* (1 × 10^7^ CFU/mL) on day 2. On day 0: All mice were inserted with silicone sheets coated with biofilm, and they were randomized into 5 groups (control, PBS), PM (blank nanoparticle), F + R, F/R@P, and F/R@PM) according to the differences in treatments. The model mice were administered 200 μL injections of PBS, PM, F + R, F/R@P, and F/R@PM solutions through the tail vein on days 3 and 7. Until the experiment was over, the mouse weight (Fig. S7) and the condition of the wound were measured and recorded every day. Fig. 6B showed macroscopic images of the wound treated over 12 days with subcutaneous implants. While there was no evident ulceration in the F/R@P and F/R@PM groups, there was apparent ulceration near the implanted site in the PBS, PM, and F + R groups, suggesting a severe bacterial infection. This study showed that NO exhibits potent antibiofilm properties through a combination of metabolic and immunological inhibition mechanisms.

On day 12, the implants were removed and subsequently examined using SEM and photographed. It was discovered that the PBS, PM, and F + R groups’ silicone sheet surfaces covered up noticeable pus, blood, and necrotic tissue. On the other hand, F/R@P and F/R@PM silicone sheets had noticeably cleaner surfaces (Fig. 6C). The results were corroborated using SEM scans (Fig. 6C), which showed that the F/R@P and F/R@PM groups had less leftover bacteria (yellow pseudo coloring) on their silicone surfaces than the PBS, PM, and F + R groups did. The plants in the F/R@PM group, on the other hand, showed the fewest bacteria that remained, demonstrating superior antibiofilm efficacy. The ultrasonic oscillation solution and subcutaneous tissue homogenates for implants produced LB results that were similar to SEM images (Fig. 6D).

On day 12, peripheral tissues from the infected areas were taken for histological examination. Images captured from hematoxylin and eosin (H&E) staining revealed a notable infiltration of inflammatory cells in the PBS, PM, and F + R groups, suggesting an inflammatory response triggered by infection. Nonetheless, H&E staining showed that the NO synergistic immunological augmented metabolic interference was responsible for the effective regulation of inflammation in the F/R@P and F/R@PM treatment groups. Interestingly, entire squamous epithelium, hair follicle, and new collagen fiber growth were shown by H&E staining on day 12 in the F/R@P and F/R@PM groups (Fig. 6E). Significant bacterial infiltration was found in the PBS, PM, and F + R groups by Giemsa staining. By contrast, the F/R@P and F/R@PM groups showed superior antibiofilm efficacy as the number of stained bacteria steadily decreased in those groups. The peripheral blood was also subjected to standard blood tests. The mice in the PBS, PM, and F + R groups had significantly increased white blood cell, neutrophil, and lymphocyte counts (*P* < 0.01); this could be because of the fact that the wounds still had biofilm infection (Fig. S8).

### 2.6 F/R@PM combat all-stage IAIs through macrophages rousing in vivo

**Fig. 7:**
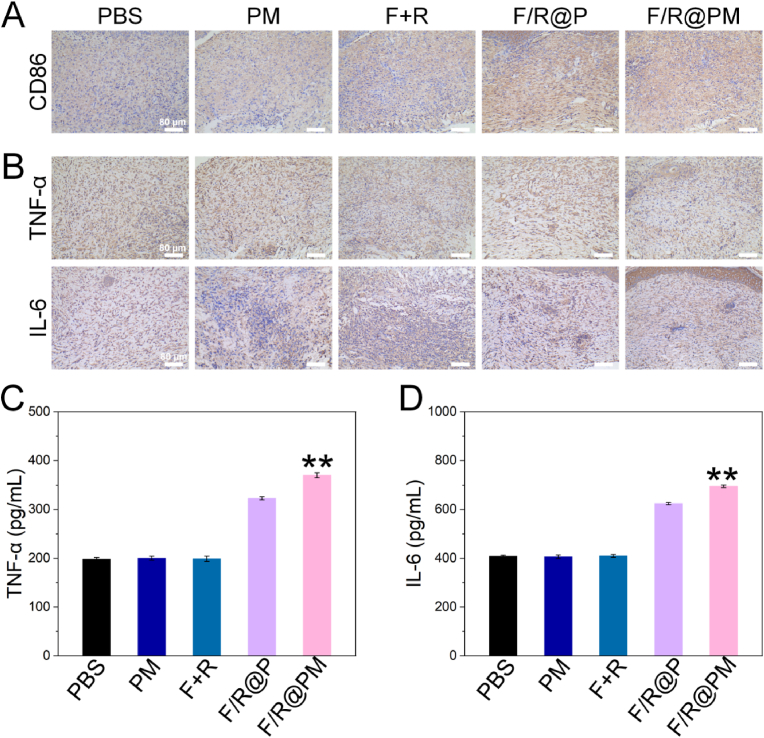
Immunomodulatory function of F/R@PM in vivo. (**A**) Immunohistochemical images of peripheral tissues stained with CD86 antibody. (**B**) Immunohistochemical images of peripheral tissues stained with TNF-*α* and IL-6 antibody. (**C**) ELISA results of peripheral tissue homogenates. (n = 3, ∗∗*P* < 0.01).

After the IAI elimination activity was successfully assessed in vivo, more research was done on F/R@PM's immunomodulatory effects. F/R@PM may promote macrophage differentiation into the M1 phenotype, as previously mentioned. Subcutaneous tissues were shown to contain CD86, a typical biomarker of the pro-inflammatory M1 phenotype of macrophages. The pro-inflammatory M1 marker CD86 levels in the subcutaneous tissues stained with immunohistochemistry (IHC) on day 12 did not significantly differ between the PBS, PM, and F + R groups. However, the F/R@P and F/R@PM groups' subcutaneous tissue showed strong CD86 signals, suggesting that R837 may promote macrophage differentiation toward the M1 phenotype, improving macrophage mobility, and restoring their capacity to kill bacteria (Fig. 7A).

Pro-inflammatory cytokines such as TNF-*α*, IL-6, and IL-1β are released by M1 macrophages, facilitating the removal of pathogenic bacteria. In addition, we measured the levels of cytokine expression using IHC. The F/R@P and F/R@PM groups have higher TNF-*α*, IL-6, and IL-1β levels, as seen in Fig. 7B. TNF-*α*, IL-6, and IL-1β expression were all lower in the PBS, PM, and F + R groups. Day 12 ELISA results of subcutaneous tissue homogenates showed that the F/R@P and F/R@PM groups had high expression levels of the pro-inflammatory factors TNF-*α* and IL-6 (*P* < 0.01, Fig. 7C and D).

### 2.7 Evaluation of biosafety of F/R@PM in vitro and in vivo

In vitro, tests were performed to determine the cytocompatibility of F/R@PM at different concentrations using HUVECs and RAW264.7 cells. When F/R@PM concentrations were lower than 400 μg/mL, 90 % of HUVECs and RAW264.7 cells were still viable (Fig. S9). For biomaterials, especially those in direct contact with blood, blood compatibility is a crucial safety criterion [37]. Consequently, we examined the in vitro blood compatibility of F/R@PM NPs. To guarantee biosafety, peripheral blood was drawn for hemolysis and biochemical testing. A range of F/R@PM concentrations—12.5, 25, 50, 100, and 200 μg/mL—was established. Less than 5 % hemolysis was observed in all groups in the final results (*P* > 0.05), indicating that the F/R@PM concentrations required for the biological experiment were satisfied (Fig. S10). Following treatment, all groups’ levels of glutathione aminotransferase, glutathione transaminase, urea, and creatinine in the biochemical test were normal (Fig. S11). Additionally, no anomalies were observed in the H&E staining images of the major organs (heart, liver, spleen, lung, and kidney) (Fig. S12), demonstrating the strong histocompatibility of the F/R@PM.

## 3 Conclusions

In conclusion, tailored to BME, we have successfully developed a macrophage-like biomimetic nanoparticle (termed as F/R@PM). Experiments demonstrated that the F/R@PM exerted an excellent ability of dispersing biofilm by disrupting dense biofilm structure due to the high penetrability of produced NO and effectively triggering local innate immunity against biofilms through inducing the polarization transition of macrophages to the M1 state. This superior synergistic effect of potent antibiofilm and long-term immunomodulation of F/R@PM has shown a high efficiency in treating IAIs. Therefore, our work suggests a novel method for treating deep IAIs by combining gas therapy with immunotherapy, which shows great promise in the field of nanomedicine anti-infection.

## 4 Experimental section

### 4.1 Materials

The Cell Counting Kit-8 (CCK-8) was acquired from Beijing Solarbio (Beijing, China). Jinan Daigang Biomaterial Co., Ltd. (Jinan, China) supplied the PLGA 50:50 Mn = 8000. BCA protein assay kit was acquired from Solarbio Technology Co., Ltd. From Beyotime Biotechnology Co., Ltd. (Shanghai, China), Hoechst 33342 was acquired. An enzyme-linked immunosorbent assay (ELISA) kit for tumor necrosis factor-alpha (TNF-α) and interleukin (IL)-6 was acquired from MultiSciences Biotech Co., Ltd. (Hangzhou, China). Dulbecco's modified eagle medium (DMEM), F12, and fetal bovine serum (FBS) were procured from ThermoFisher Biochemical Products (Beijing) Co., Ltd. (Beijing, China). As per our previous report, NO donor FOT was synthesized [23]. All other chemicals, unless otherwise specified, were of analytical grade and were acquired from SinoPharm Chemical Reagent Co., Ltd. (Shanghai, China). The chemicals were utilized directly without any additional purification.

### 4.2 Cell lines and animals

Mouse mononuclear macrophage leukemia cell line RAW264.7 and human umbilical vein endothelial cells line (HUVEC) were obtained from Southwest Jiaotong University (Chengdu, China) and cultured in DMEM high glucose medium and F12 medium, respectively, both of which contained 10 % FBS, at 37 °C in a 5 % CO_2_ humidified environment incubator (Thermo Scientific, Sunnyvale, CA).

The Institutional Animal Care and Use Committee of Sichuan University (Approval Number: 20231103010) approved the use of female BALB/c mice (6–8 weeks) at 25 °C and 55 % humidity. All animal experiments were conducted following established protocols.

### 4.3 Isolation of RAW264.7 cell membrane

Protease inhibitor-containing cell lysis buffer was used to scatter the RAW264.7 cells. After 30 min in an ice bath with an ultrasonic cell breaker, the RAW264.7 cells were separated into their constituent parts and their organelles by centrifuging the supernatant at 10,000 g for 60 min. RAW264.7 cell membrane was obtained by collecting the supernatant and spinning it at 100,000 g for 120 min. This membrane was then used as a precipitate in the centrifuge tube. The total protein content in the obtained macrophage membrane was examined using the bicinchoninic acid (BCA) protein assay.

### 4.4 Preparation of F/R@PM

A total of 10 mL of acetone was used to dissolve FOT (2 mg), R837 (1 mg), and PLGA (20 mg). The mixed solution was slowly added to 10 mL of 1 % (w/v) polyvinyl alcohol deionized water, and it was agitated at room temperature for 24 h to produce NPs and evaporate acetone. Using ultrafiltration, FOT and R837-loaded PLGA NPs (F/R@P) were gathered. At a protein-to-polymer ratio of 1:1 (w/w), the gathered F/R@P was mixed with RAW 264.7 cell membrane and sonicated for 3 min at 4 °C in a sonicator bath (SHUNMATECH SM-900A, 25 kHz, 100 W). The F/R@PM NPs were then created by extruding the mixture through a 200 nm polycarbonate barrier. The procedure used to prepare the PLGA (P) and RAW 264.7 cell membrane-coated PLGA (PM) NPs was the same.

### 4.5 Characterization of the NPs

Using a Zeta-sizer, Nano-ZS90 (Malvern Instruments, Malvern, Worcestershire, United Kingdom), the hydrodynamic diameter and zeta potential of several NPs were determined at 25 °C using DLS. Several nanoparticles’ morphologies were observed utilizing a transmission electron microscope (HT7700, Hitachi Electronics, Japan). FOT and R837 loading capacity were measured using an Agilent EC-C18 column (4 μm, 4.6 mm × 150 mm) with detection wavelengths of 254 and 318 nm, respectively, using high-performance liquid chromatography (HPLC) (1260, Agilent Technologies, USA).

### 4.6 Characterization of proteins

Polyacrylamide gel electrophoresis (PAGE) was used to analyze the membrane proteins sodium dodecyl sulfate (SDS)-PAGE. Using kits for cell total protein extraction, the membrane proteins of the macrophage membranes, PM, and F/R@PM were extracted. Bio-Rad electrophoresis equipment was used to run the extracted membrane proteins on a 4%–12 % Bis-Tris 10-well minigel in a running buffer. The system was operated at 75 V for 0.5 h and 140 V for 1 h. Ultimately, SimplyBlue was used to dye the resulting polyacrylamide gel for visual purposes overnight.

The integrin α4, β1, and CD47 contents in RAW 264.7 cells, RAW 264.7 cells membrane, PM, and F/R@PM were measured by western blot analysis. The total protein of the lysis solution from RAW 264.7 cells, RAW 264.7 cells membrane extracted from 1 × 10^7^ cells, and the subsequent PM and F/R@PM were extracted by Cell Total Protein Extraction kits and used for measurements. Following electrophoresis on a 10 % SDS-PAGE, samples were transferred using a polyvinylidene difluoride membrane (Millipore, USA). The membranes were previously treated with primary antibodies against α4 (anti-integrin α4, 8440S, CST), β1 (anti-integrin β1, 34971, CST), and CD47 (anti-CD47 antibody, ab175388, Abcam). This was followed by the addition of horseradish peroxidase-labeled goat/rabbit IgG (H + L) from Beyotime, Jiangsu, China. The protein signals were measured using an imaging system called a ChemiDoc MP (Bio-Rad, USA), with the assistance of enhanced chemiluminescence.

### 4.7 Drug loading and in vitro drug release study

The F/R@PM lyophilized powder was dissolved in CH_3_OH, and an HPLC set up at 254 nm and 318 nm, respectively, was used to measure the contents of FOT and R837. The previously defined standard curves for FOT and R837 in CH_3_OH were used to calculate the drug loading efficiency (LE) and drug encapsulation efficiency (EE).

Using a dialysis method, the releases of FOT and R837 from F/R@PM were examined separately. To sum up, disposable dialysis bags (MWCO: 3500 Da) were filled with 1 mL of F/R@PM solutions (2 mg/mL). The dialysis bags were then immersed at 37 °C in 50 mL of phosphate-buffered saline (PBS) solution (release medium, pH 7.4 or 6.5). One milliliter of release medium was collected for analysis at predetermined intervals, and it was replaced with an equivalent volume of fresh PBS at 37 °C. HPLC was used to measure the total quantity of FOT and R837 emitted at 254 and 318 nm, respectively.

### 4.8 In vitro NO detection

The amount of NO release from F/R@PM was measured using the Griess Reagent (Beyotime, Shanghai, China). Briefly, 200 μg of F/R@PM was spread out across the tube using 1.0 mL of ABS buffer (pH 5.0), either with or without GSH. Following varying periods of shaking at 37 °C (1, 6, 12, and 24 h), 100 μL of the solution was transferred to a 96-well plate. After that, the well plate was filled with 50 μL of Griess Reagent I and 50 μL of Griess Reagent II, followed by incubation for 5 min. Using a microplate reader, the mixed solution's optical density (OD) was measured at 540 nm. With the use of a standard curve established using NaNO_2_ solutions with known concentrations, the amount of NO release was calculated.

### 4.9 Bacterial culture

We transferred *S. aureus* on solid Luria-Bertani (LB) agar plates to 3 mL of liquid LB culture medium and grew them for 16 h in a shaking incubator at 37 °C with 120 rpm rotation. We added the liquid LB culture medium to the bacteria before using them again, and the OD value at 600 nm was 0.1. We divided *S. aureus* into 4 treatment groups (PBS, FOT + R837 (F + R), F/R@P, and F/R@PM) using a standard antibacterial assay. Each tube had a total volume of 1.0 mL of solution. We diluted the bacterial suspension appropriately after 12 h of incubation. Subsequently, 30 μL of the remaining bacterial cells were spread onto the agar culture plate and inoculated at 37 °C for 24 h.

### 4.10 Morphological observation of the bacteria

Following a variety of treatments, the bacteria were re-dispersed in 1 mL of 2.5 % glutaraldehyde after being centrifuged for 5 min at 4000 rpm. The fixed groups were administered ethanol concentrations ranging from 10 % to 90 % and were gradually dehydrated for 30 min after the 12 h. Samples of bacteria were spread out in 100 μL of ethanol and arranged on a cooper grid for SEM (JEOL JSM-IT700HR, Japan) examination.

### 4.11 Biofilm inhibition assay

The F + R, F/R@P, and F/R@PM were placed in a 96-well plate and co-cultured with *S. aureus* for 24 h, respectively. While the group without any treatment was regarded as control group. Next, supernatants were transferred to another 96 well plate and analyzed using a wavelength of 490 nm. After that, the samples were stained with SYTO 9/PI (6 μmol/L) for 15 min. Then the samples were observed using fluorescence microscope microscopy (FM) (Olympus IX-73, Japan). An alternative method involved aspirating the liquid from the 96-well plate, fixing the biofilm at the bottom of the plate with anhydrous ethanol, and then staining it with crystal violet for 20 min. After discarding the staining solution, a digital camera was used to capture an image of the stained biofilm. To assess the biomass of the biofilm, the biofilm was re-solubilized in 30 % ethylic acid, and its absorbance at 550 nm was measured using an enzyme-labeled microplate reader (Molecular Devices 1712, USA).

### 4.12 In vitro cellular uptake

The cellular uptake of NPs was detected using flow cytometry and FM. RAW264.7 and HUVEC cells (5 × 10^4^) were seeded in 6-well plates for 24 h. After the cells adhered to the wall and were treated with a medium containing RB-loaded P NPs (RB@P) and RB-loaded PM NPs (RB@PM) for 3 and 6 h. After washing three times with PBS, the cells were collected and analyzed using flow cytometry. The excitation wavelength was 546 nm and the emission wavelength was 568 nm (BD Accuri C6, USA). RAW 264.7 and HUVEC cells (1 × 10^5^) were seeded in 6-well plates for 24 h. After the cells adhered to the wall and were treated with a medium containing RB-loaded P NPs (RB@P) and RB-loaded PM NPs (RB@PM) for 3 and 6 h. The cells were washed 3 times with PBS and stained with Hoechst for 10 min. After washing 3 times with PBS, the cells were observed using FM (Leica DMi8, Germany).

### 4.13 Immunomodulatory experiments in vitro

RAW264.7 cells were seeded in 6-well plates at a density of 2 × 10^5^ cells per well. After incubating the media for 1 day, we added a new medium containing F + R, F/R@P, or F/R@PM. We retained the treated RAW 264.7 cells for further analysis after a 12-h incubation period. We collected the medium and extracted the cell debris and NPs by centrifuging it for 20 min at 4 °C and 3000 rpm. ELISA kits were utilized to measure the levels of TNF-*α* and IL-6 in the resultant medium following the guidelines provided by the manufacturer.

We scratched RAW264.7 cells from the plate surfaces and transferred them into centrifuge tubes for the flow cytometry experiment. After centrifuging, cleaning, and re-suspending the cells in each group in 100 μL PBS containing 0.25 μg of APC-conjugated anti-mouse CD86 antibody (Biolegend, USA), they were incubated for 30 min at 4 °C in the dark. Finally, we assessed the expression of CD86 on RAW 264.7 cells using flow cytometry.

### 4.14 Functional evaluation of macrophages

We examined the phagocytic activity of RAW264.7 cells. In short, 2 × 10^5^ cells per well of 6-well plates were sown with RAW264.7 cells. After a day of incubation, we replaced the medium with a new one that contained F + R, F/R@P, or F/R@PM. We harvested the cells and processed them into a cell suspension after a 12-h incubation period. After that, a 6-well plate containing sample-treated cell suspension was filled with pre-prepared bacterial biofilm suspension cell suspension, and the plates were co-incubated for 2 h at 37 °C. To eliminate extracellular bacteria, the suspension was then replaced with new DMEM containing 200 g/mL of gentamicin and incubated for an additional hour at 37 °C. After discarding the media, the cells underwent washing and fixation for transmission electron microscopy (TEM) observation of the internal bacteria. After removing the gentamicin medium, we added 1 mL of 1 % Triton X-100 into each well to penetrate the macrophages' cell membrane and induce engulfed bacteria leakage. We collected the Triton X-100 solution to enumerate the bacteria using the gradient dilution and spreading plate method. We counted the colonies after incubation for 24 h.

### 4.15 Preparation of silicone sheets

Briefly, silicone was initially sliced into 8 mm diameter and 0.5 mm thick sheets. After being sterilized, the silicone square sheets were transferred to a 6-well plate. After that, the plate was filled with 2 mL of *S. aureus* bacterial suspension (107 CFU/mL). The plate was then incubated for 2 days at 37 °C with 5 % CO_2_ to grow bacterial biofilm. Every 24 h, a fresh medium was added to the culture. The silicone sheets were collected 2 days later and utilized to establish the mouse BAI model.

### 4.16 Mouse IAIs model

We randomly assigned 40 BALB/c mice each into five groups: PM, F + R, F/R@P, F/R@PM, and control (PBS). We evaluated the in vivo anti-biofilm capacities using mouse biofilm-associated infection models. We anesthetized the mice with 1 % pentobarbital sodium before shaving, cleaning, and cutting their backs. Following subcutaneous implantation of the biofilm-produced silicone sheets, any skin incisions were promptly sutured. Mice in the “treated groups” received 200 μL of F + R, F/R@P, or F/R@PM solution on days 3 and 5. The untreated group received an injection of PM or PBS in an equivalent volume. We captured the affected area on days 1, 5, 10, and 12 following injection. We sacrificed some animals on day 7, added a dose of CO_2_, and removed soft tissues to assess the treated groups’ ability to combat all-stage IAIs in vivo through macrophage re-stimulation. We killed the remaining mice on day 12.

### 4.17 Targeting of PM NPs in vivo

The IAIs mouse model was generated as described above. After 1 day, the mice were intravenously injected with PBS free RD, RD-loaded P NPs and MP NPs, respectively. After 24 h post-injection, the silicone sheets and major organs (hearts, livers, spleens, lungs, and kidneys) were harvested from the mice, and subsequently, ex vivo imaging was acquired using in vivo imaging systems (IVIS) (PerkinElmer, USA).

### 4.18 In vivo immunoregulatory assay

Soft tissue slices were prepared and used for immunohistochemical (IHC) staining. For IHC analysis, tissue slices were incubated with the primary antibodies anti-CD86 Rabbit pAb (1:100, Bioss), TNF-α Rabbit pAb (1:100, Servicebio), and IL-6 Rabbit pAb (1:100, Servicebio) for 12 h, followed by secondary antibody HRP-conjugated goat anti-rabbit IgG ([H + L], 1:100, Servicebio) for 0.5 h. The diaminobenzidine detection kit was used to visualize the binding sites. All stained slices were observed under a microscope.

### 4.19 Biosafety evaluation

The heart, liver, spleen, lung, and kidney were extracted for H&E staining. Specifically, mouse blood samples were collected to conduct a comprehensive blood count and assess serum biochemical indicators.

### 4.20 Histological analysis

We collected the soft tissues surrounding the implants, fixed them with 4 % paraformaldehyde, and dehydrated them with serial alcohol solutions. We then performed paraffin wax embedding and slicing. After staining the sections with H&E (Servicebio, Wuhan, China), we evaluated the pathological alterations in each group using optical microscopy.

### 4.21 Statistical analysis

All data were represented as the mean ± standard deviation. The experimental sample count was three (n = 3). The two-sided Student's t-test and one-way analysis of variance were employed to compare the differences between and among the categories. *P*-value<0.05 were deemed to be statistically significant.

## CRediT authorship contribution statement

**Guoqing Wei:** Writing – original draft, Supervision, Project administration, Methodology, Investigation, Funding acquisition, Formal analysis, Data curation. **Tiantian Xiao:** Writing – original draft, Software, Methodology, Formal analysis, Data curation. **Yufeng Xi:** Writing – original draft, Methodology, Formal analysis, Data curation. **Rong Ju:** Writing – original draft, Supervision, Funding acquisition, Formal analysis.

## Data sharing statement

The data that supports the findings of this study are available from the corresponding author upon reasonable request.

## Declaration of competing interest

The authors declare that they have no known competing financial interests or personal relationships that could have appeared to influence the work reported in this paper.

## Data Availability

Data will be made available on request.
