# Supplementary material for: A macrophage-like biomimetic nanoparticle with high-efficiency biofilm disruption and innate immunity activation for implant-related infection therapy
Source: Mater Today Bio. 2025 Feb 14;31:101575. doi: 10.1016/j.mtbio.2025.101575 (PMC11883385; doi:10.1016/j.mtbio.2025.101575)
Supplement: Multimedia component 1 [file mmc1.docx]

*Supplementary materials*


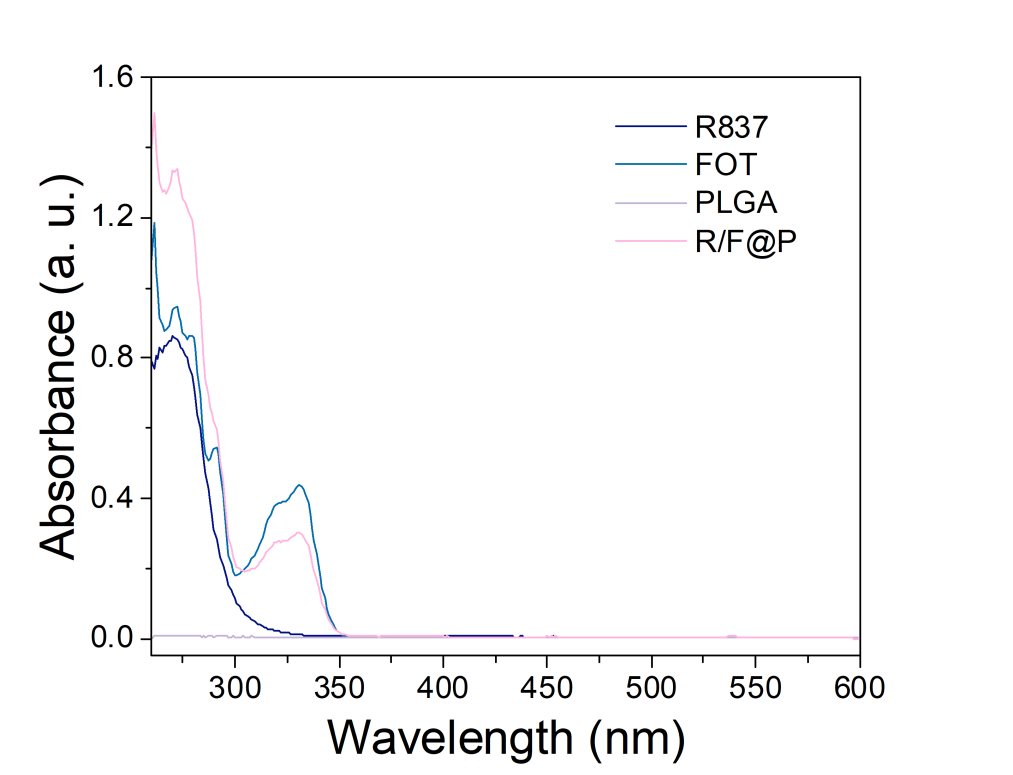


**Fig. S1**. The Ultraviolet-visible absorption spectrum of different samples in DMSO.


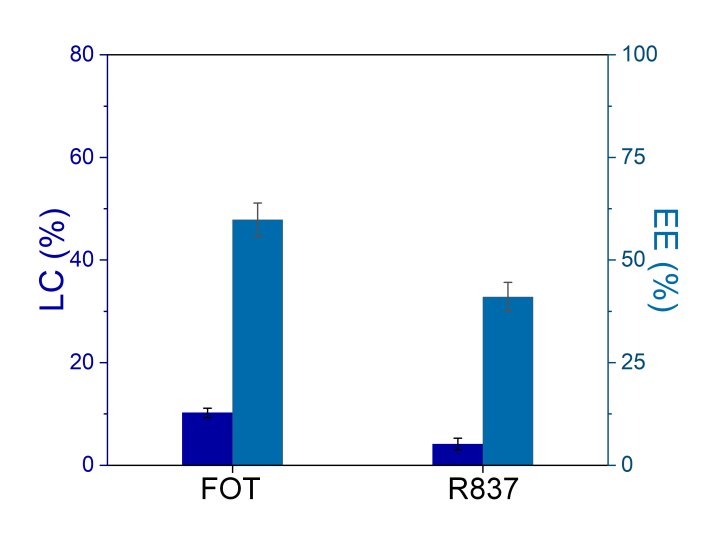


**Fig. S2**. Loading and encapsulation efficiency of F/R@PM (n=3).


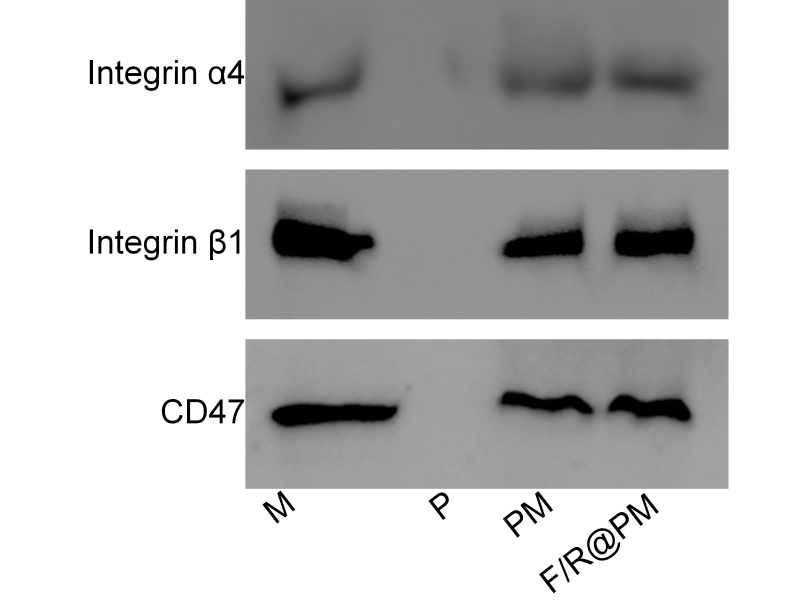


**Fig. S3**. Representative Western blots of membrane proteins in fresh macrophage cell membrane (M), P, PM, and F/R@PM.


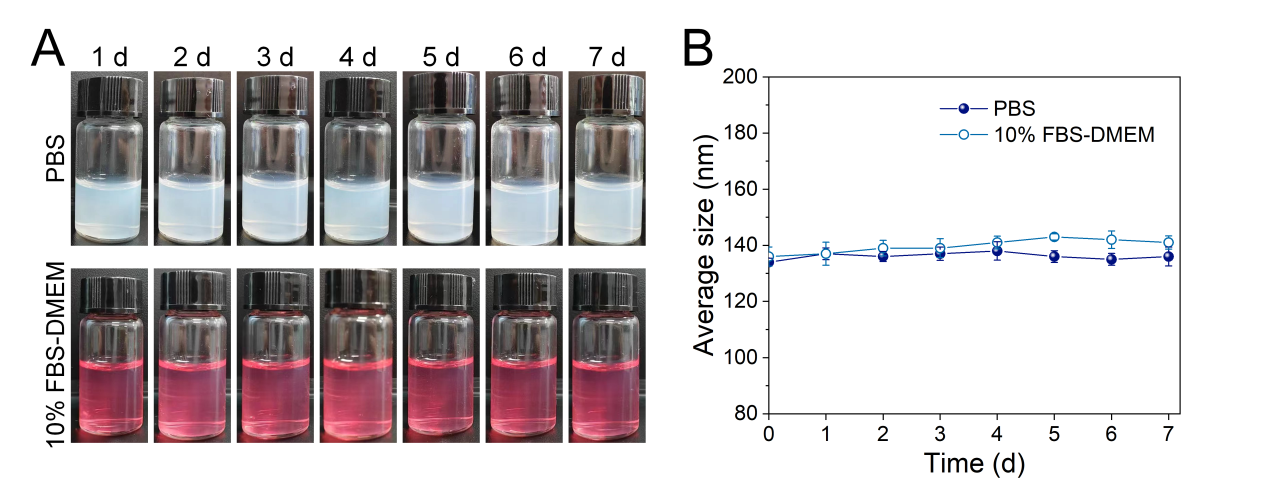


**Fig. S4**. Stability assessment of F/R@PM NPs in PBS and 10% FBS-DMEM during 7 d. (A) Physical photographs. (B) The curve chart of the average size change (n=3).


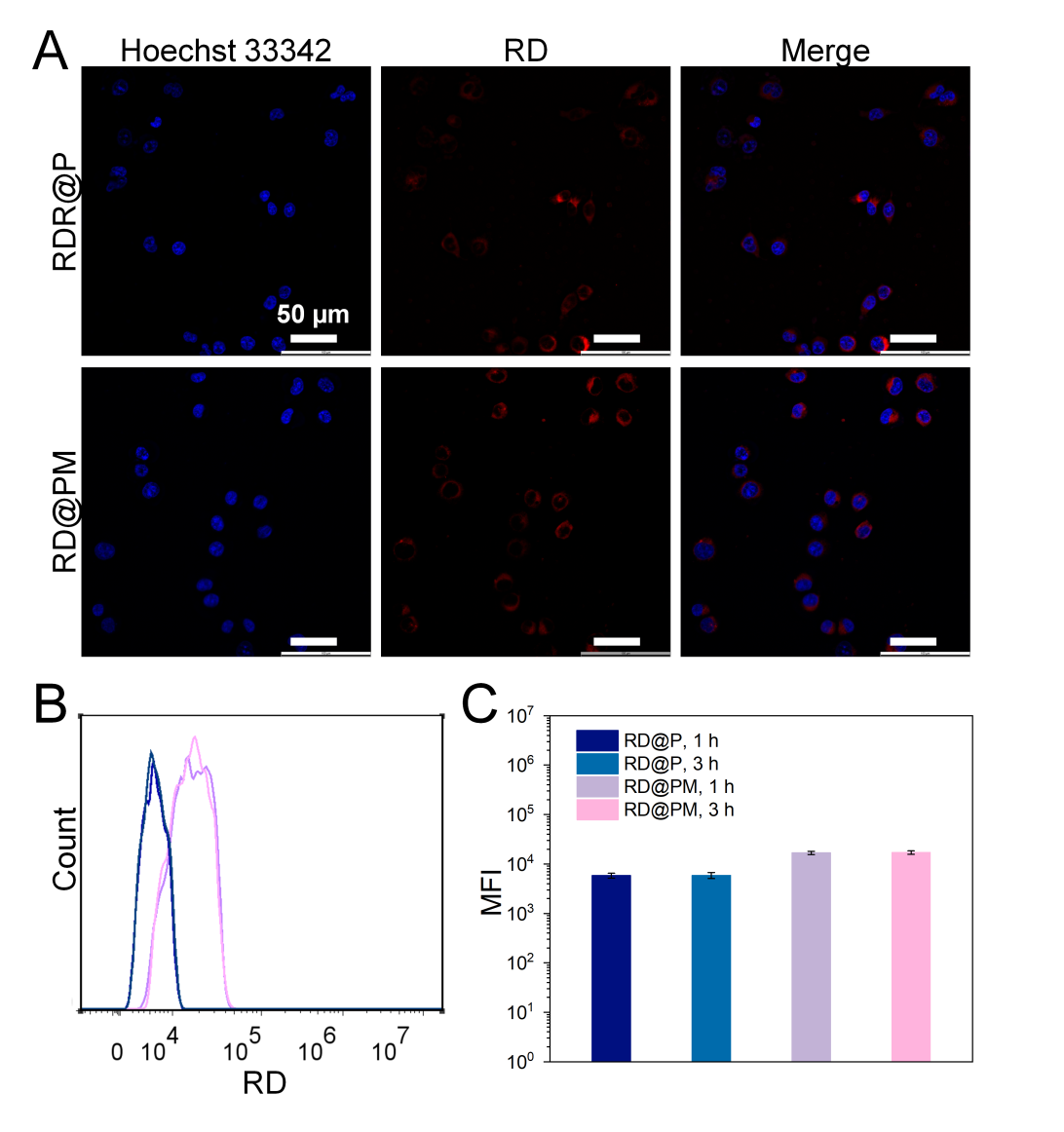


**Fig. S5**. (A) Representative fluorescence images of NR@P and NR@PM internalized HUVEC cells. (B) FACS results of cellular uptake of NR@P and NR@PM in HUVEC cells. (C) Quantification of cellular uptake of NR@P and NR@PM in HUVEC cells (n = 3).


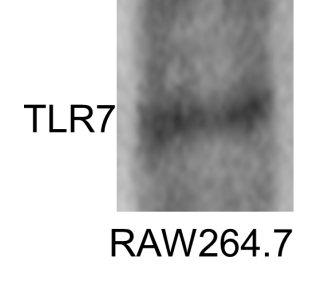


**Fig. S6**. Western blotting analysis of the protein expression levels of TLR7 of RAW264.7 cells.


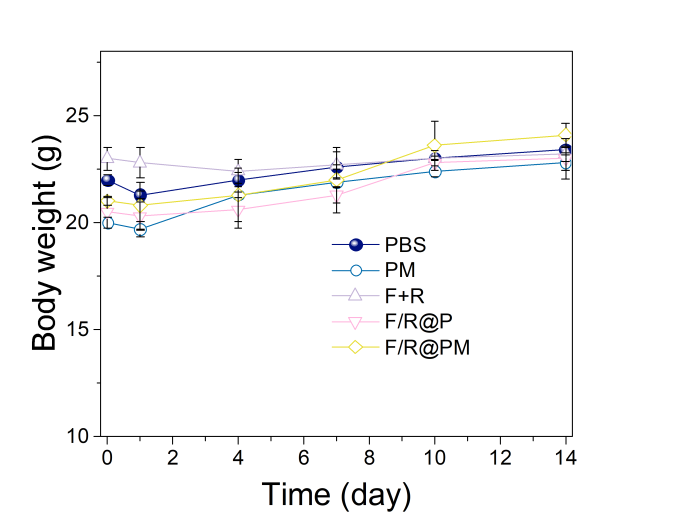


**Fig. S7**. Relative body weight of the mice with various treatments.


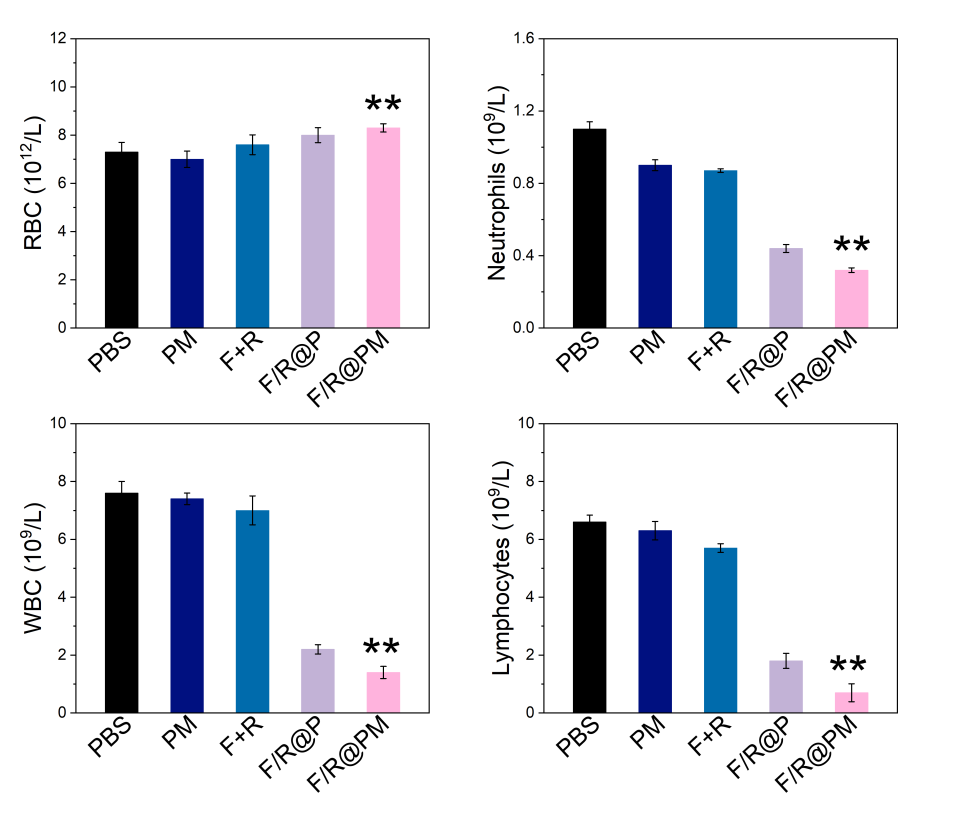


**Fig. S8**. The peripheral blood cell counts of each mouse in all groups (n = 3, ***P* < 0.01). Normal value reference range: RBC: 6.36-9.42(10^12^/L); WBC: 0.8-6.8(10^9^/L); Lymphocytes count: 0.7-5.7(10^9^/L); Neutrophils count: 0.1-1.8(10^9^/L).


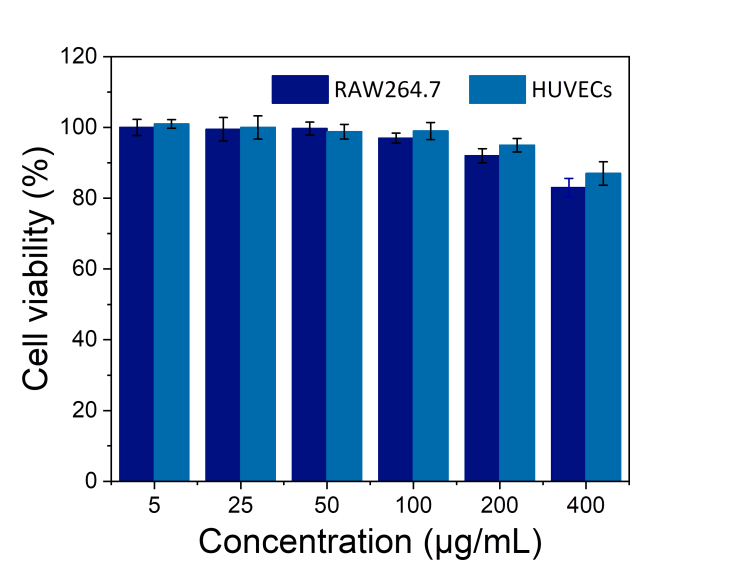


**Fig. S9**. Cell viability of RAW264.7 and HUVEC cells after incubation with various doses of F/R@PM NPs for 24 h (n =3).


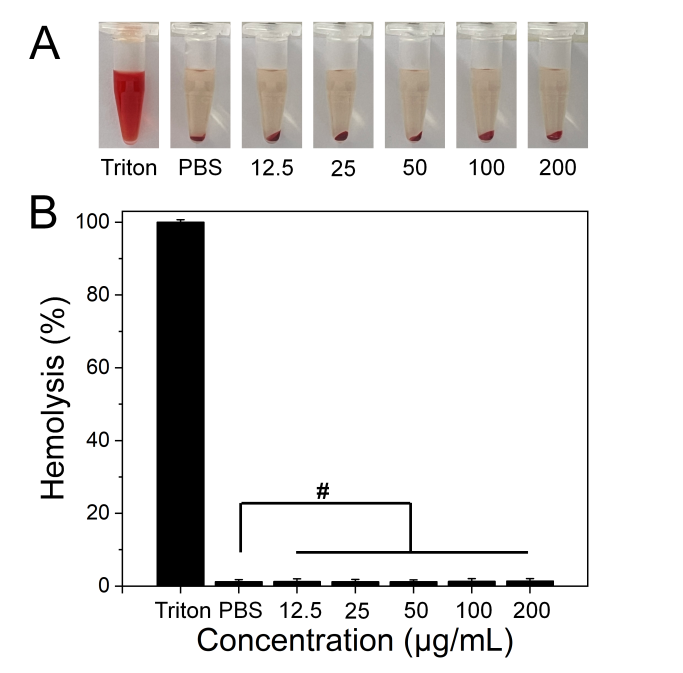


**Fig. S10**. Hemolysis photographs (A) and hemolysis rate (B) of blood cellsincubated with different treatments (n = 3, ^#^*P* > 0.05) .


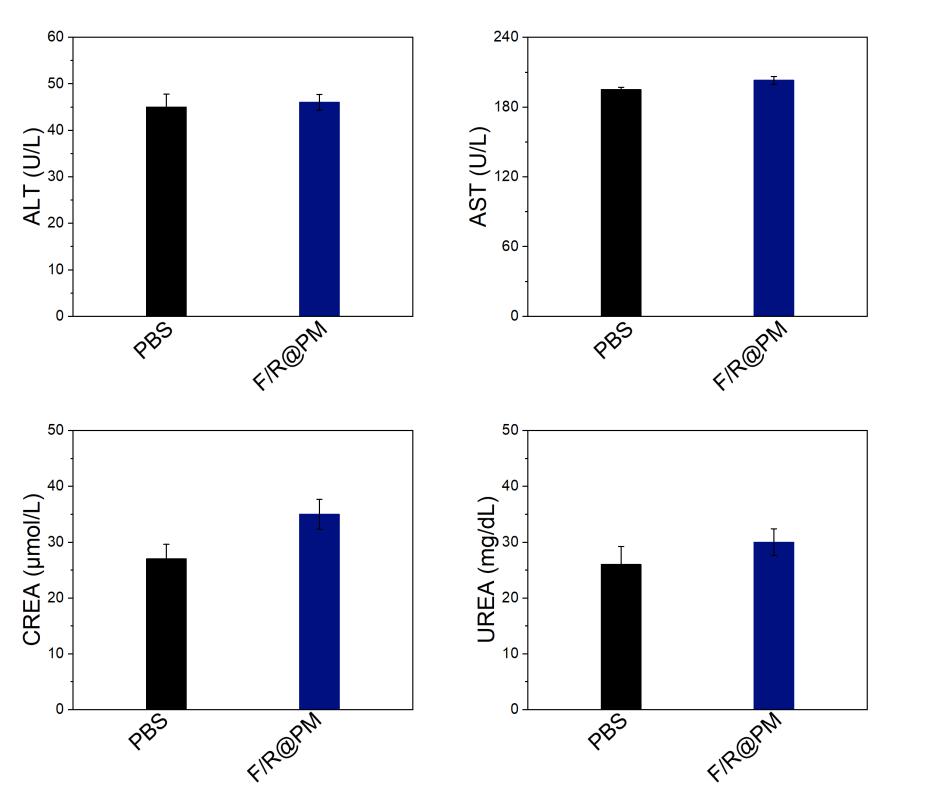


**Fig. S11**. ALT, AST, CREA and UREA levels in circulating serum of mouse after 12 days of treatment in different groups. Normal value reference range: AST: 36.31- 235.48 U/L; ALT: 10.06-96.47 U/L; UREA: 10.81-34.74 mg/dL; CREA: 10.91-85.09 μmol/L.


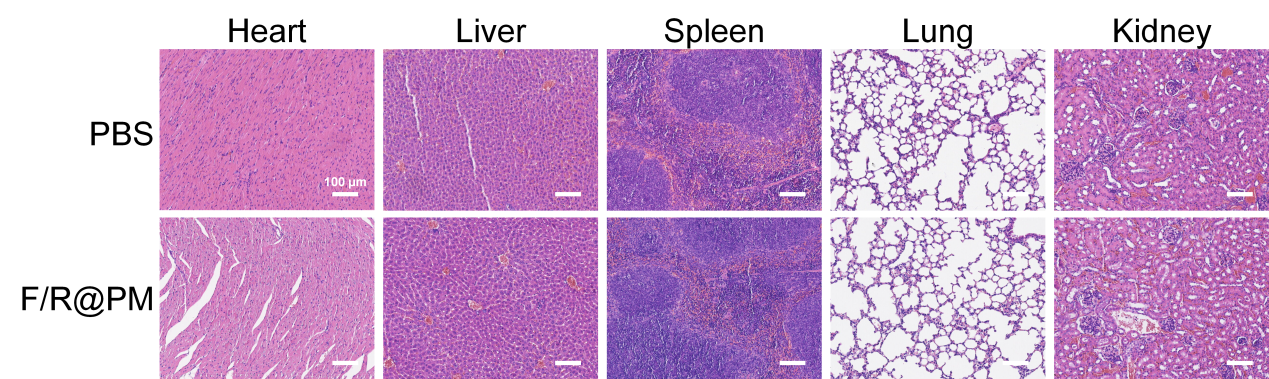


**Fig. S12**. H&E staining of organ samples from PBS and F/R@PM treatment groups.
